# The KIND Challenge community intervention to reduce loneliness and social isolation, improve mental health, and neighbourhood relationships: an international randomized controlled trial

**DOI:** 10.1007/s00127-024-02740-z

**Published:** 2024-08-19

**Authors:** Michelle H. Lim, Alexandra Hennessey, Pamela Qualter, Ben J. Smith, Lily Thurston, Robert Eres, Julianne Holt-Lunstad

**Affiliations:** 1 Prevention Research Collaboration, University of Sydney, Camperdown, NSW Australia; 2 Iverson Health Innovation Research Institute, Swinburne University of Technology, Hawthorn, Australia; 3 Manchester Institute of Education, University of Manchester, Manchester, UK; 4 Neurodisability and Rehabilitation, Murdoch Children’s Research Institute, Royal Children’s Hospital, Parkville, VIC Australia; 5 Department of Paediatrics, The University of Melbourne, Parkville, VIC Australia; 6 Department of Psychology, Neuroscience Center, Brigham Young University, Provo, UT USA

**Keywords:** Community interventions, Loneliness, Mental health, Neighbourhood relationships, Randomized controlled trial

## Abstract

**Purpose:**

Loneliness and social isolation are risk factors for poor health, but few effective interventions are deployable at scale. This study was conducted to determine whether acts of kindness can reduce loneliness and social isolation, improve mental health, and neighbourhood social cohesion.

**Method:**

Three randomized controlled trials (RCTs) were conducted in the USA, UK, and Australia, involving a total of 4284 individuals aged 18–90 years old, randomized to the KIND challenge intervention or a waitlist control group. Participants allocated to the intervention were asked to do at least one act of kindness per week within a four-week period. The primary outcome was loneliness and secondary outcomes included measures of social isolation, mental health, and neighbourhood social cohesion.

**Results:**

There was a significant, albeit small, intervention effect after four weeks for reduced loneliness in the USA and the UK, but not for Australia. Relative to controls, KIND challenge participants also showed significantly reduced social isolation and social anxiety in the USA, and reduced stress in Australia. There was also reduced neighbourhood conflict in the USA, increased number of neighbourhood contacts in the USA and Australia, greater neighbourhood stability and feelings of neighbourhood importance in the UK, and better neighbourhood social relationships in Australia.

**Conclusion:**

Promoting the provision of social support through small acts of kindness to neighbours has the potential to reduce loneliness, social isolation and social anxiety, and promote neighbourhood relationships, suggesting a potential strategy for public health campaigns.

**Trial registration:**

Clinical Trials Registry. NCT04398472. Registered 21^st^ May 2020.

**Supplementary Information:**

The online version contains supplementary material available at 10.1007/s00127-024-02740-z.

## Introduction

The health impacts and risk factors associated with loneliness and social isolation are well-established [1, 2]. There is, however, mixed and weaker evidence in regard to interventions aimed at fostering social connection, or reducing loneliness and social isolation [3]. Systematic reviews and meta-analyses of interventions in clinical settings have been effective in reducing loneliness [4, 5] and increased survival among medical patients [6]. However, there is significant heterogeneity in the degree of effectiveness. Among the interventions found to be effective, the effect sizes are often small [7], and they are often both time and resource-intensive, requiring highly trained professionals, staff, or volunteers [8]. Even prior to the COVID-19 pandemic, resources and availability of healthcare professionals and staff were limited, and these demands have been compounded since the onset of the pandemic. Thus, there is a need for effective interventions outside the clinic that can easily be adopted by the general population.

One way to address loneliness and social isolation is through fostering strong community relationships [9]. Community-based interventions targeting loneliness and social isolation are widely implemented but few are rigorously tested because many service delivery providers may face barriers such as a lack of expertise, time, or logistics. Social networking online is now a common method of connecting and engaging others within a community. While some social technology usage may exacerbate loneliness [10], digital tools that are designed to augment or facilitate connecting people offline may hold promise in both nonclinical [11, 12] and hard to engage clinical samples [13, 14]. Digital tools can be used to deliver effective strategies focused on improving relationships, for example, showing acts of kindness, has been shown to improve relationship ties within an online platform [15].

Increasingly there is interest in understanding whether using strengths-based strategies such as kindness may improve relationship quality. While a meta-analysis of experimental studies examining effects of performing acts of kindness demonstrate effectiveness on general measures of well-being [16], the impact of kindness as a stand-alone strategy to reduce loneliness has yet to be examined. There is evidence from smaller studies that acts of kindness can promote wellbeing [17], enhance relationship quality and relational functioning [18], and improve feelings of social support and happiness [19]. Therefore, performing acts of kindness towards others has the potential to reduce feelings of loneliness by minimising social isolation and improving relationship ties and satisfaction. The examples of acts of kindness parallel key elements of social support—emotional, tangible, informational, and belonging support. Studies demonstrating the benefits of social support provision on psychological well-being, building social capital, self-esteem, and health, and reducing self-focus, are plausible contributors to lowering loneliness [20].

Thus, we designed an intervention called the KIND challenge and conducted a randomized controlled trial (RCT) to evaluate the impact of kindness on reducing loneliness and social isolation, improving mental health outcomes, and promoting neighbourhood relationships in three countries (USA, UK, and Australia).

## Method

This study was approved by Swinburne University of Technology, University of Manchester, and Brigham Young University’s institutional Ethics Review Boards. CONSORT reporting guidelines were followed. The evaluation of the KIND Challenge was pre-registered at Clinical Trials (ClinicalTrials.gov Identifier: NCT04398472) prior to the study launch, and a protocol paper has been published [21].

### Trial design

Three RCTs were conducted, one in each country (USA, UK, and Australia) in July–September 2020. Each RCT had two arms: the KIND Challenge and waitlist control. Those randomized to the KIND Challenge were asked to complete at least one act of kindness per week for four weeks; those randomized to the waitlist condition continued their typical practice during this time. Baseline assessment was conducted pre-randomization and follow-up assessment four weeks later following the completion of the intervention.

### Sample recruitment, characteristics, and randomization

**Fig. 1:**
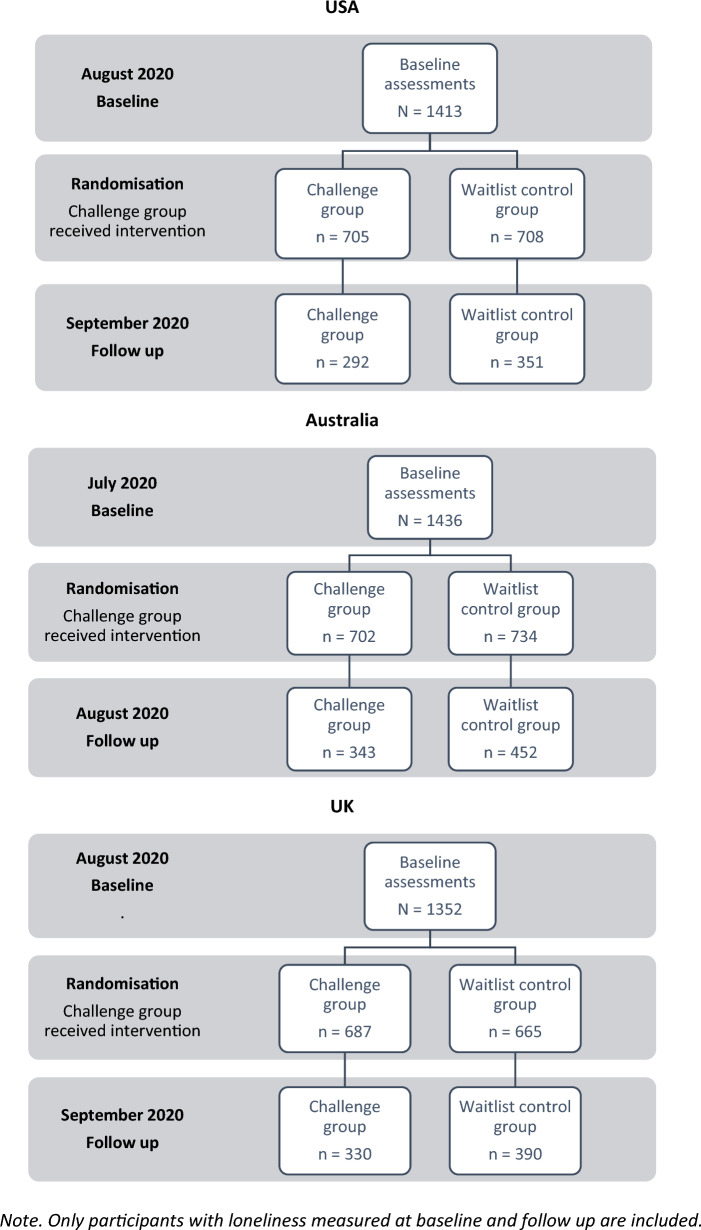
Recruitment flow chart across three countries. Only participants with loneliness measured at baseline and follow up are included

Community participants aged 18–90 years, who were users of an online social networking platform (Nextdoor), were invited to commit to the challenge for four weeks. First, a brief study recruitment advertisement was shown to them through the Nextdoor platform. Those interested clicked to see the participant information form for more details. People who were interested consented online and were sent the first survey link. Exclusion criterion included individuals without proficient English reading comprehension skills. The target sample size was 1452 participants from each country (see supplementary table data 4 for post hoc sample size calculations). After consenting to the study, participants completed the baseline survey and then were randomized to either the KIND Challenge or waitlist control. Those who consented and allocated to the KIND Challenge were only told to perform acts of kindness after they complete the first baseline survey. A total of 4284 participants were recruited between July and September 2020 and eligible for randomization into their country’s trial. The study was closed after minimum sample size was recruited. See Fig. 1 for the recruitment flow chart and Table 1 for sample sizes and demographic characteristics across intervention arms and country.

**Table 1:** Demographics of sample by country and trial allocation

	USA		Australia		UK	
	KIND Challenge	Waitlist	KIND Challenge	Waitlist	KIND Challenge	Waitlist
*N*	711	712	709	742	708	702
Age mean (range) in years	49 (18–89)	50 (18–85)	60 (19–90)	60 (19–89)	55 (18–90)	55 (18–89)
Gender % (n)						
Female	89.2% (634)	85.5% (609)	64.0% (454)	67.3% (499)	73.3% (519)	73.9% (519)
Male	9.7% (69)	12.9% (92)	35.4% (251)	31.9% (237)	25.1% (178)	25.5% (179)
Other	1.1% (8)	1.5% (11)	0.6% (4)	0.8% (6)	1.6% (11)	0.6% (4)
How long have you lived in your neighbourhood?						
Less than 1 year	15.0% (107)	17.4% (124)	9.6% (68)	8.5% (63)	11.8% (83)	9.5% (66)
1–5 years	39.8% (283)	36.0% (256)	29.3% (208)	31.4% (233)	27.8% (196)	26.7% (186)
6–10 years	13.9% (99)	14.9% (106)	15.5% (110)	13.8% (102)	13.6% (96)	15.2% (106)
Over 10 years	31.2% (222)	31.7% (226)	45.6% (323)	46.3% (343)	46.8% (330)	48.6% (338)
Nextdoor membership length						
Less than 1 month	6.3% (45)	6.2% (44)	16.2% (114)	13.8% (102)	18.8% (131)	20.9% (143)
Less than 6 months	17.3% (123)	18.8% (134)	27.9% (197)	28.9% (213)	24.7% (172)	25.1% (172)
Less than 1 year	15.7% (111)	16.6% (118)	23.9% (169)	24.5% (181)	18.7% (130)	18.4% (126)
Between 1–5 years	49.1% (348)	48.5% (345)	29.9% (211)	30.5% (225)	33.2% (231)	31.8% (218)
More than 5 years	11.6% (82)	9.8% (70)	2.1% (15)	2.3% (17)	4.5% (31)	2.8% (26)
Perceived social restrictions (0–12) mean (SD) at baseline	5.06 (2.47)	5.14 (2.48)	7.13 (2.95)	7.04 (3.03)	4.10 (2.50)	4.02 (2.52)

Simple randomization (1:1) was used because the study was conducted entirely on the online questionnaire software Qualtrics. Blinding participants to their assigned condition was not possible due to the nature of the intervention; however, research personnel were blind to the condition until after data was collected.

### Intervention

Participants randomized to the KIND challenge intervention arm were encouraged to perform self-selected kindness activities over four weeks (see Table 2 for an overview of types of kind acts suggested). Because the trial took place during the COVID-19 pandemic, participants were asked to adhere to their state or country’s health department safety guidelines during the KIND challenge.

**Table 2:** KIND Challenge tasks and support types

Support type	Tasks
Emotional support	Show care and concern for a neighbor (e.g., provide a listening ear, cheer up a neighbour who is down, check in on the welfare of a neighbour)
Informational support	Provide advice or helpful information to a neighbor (e.g., where to shop, good doctors in the area, potential job opportunities, gardening tips, etc.)
Tangible support	Help a neighbour out (e.g., mow their lawn, take in their garbage bins, offer meals, run errands, bring them groceries, etc.)
Belonging support	Contribute to a larger neighbourhood effort, action, or activity (e.g., support a neighbourhood business, share your talents/skills with others, neighbourhood clean-up, volunteering, etc.)
Companionship support	Have regular contact with a neighbor (e.g., chat across the fence, street, or balcony, call on the phone, etc.)

The KIND Challenge activities were selected because they are considered to be positive, engaging, and feasible to the average individual, and draw upon evidence concerning the benefits of emotional support, informational support, tangible support, belonging support [22], and companionship [23–25]. Participants in the KIND Challenge arm received an email reminder to complete the activity weekly for the four weeks of the intervention.

### Measures

All measures were administered through the online survey platform Qualtrics. The primary outcome, loneliness, was measured using the UCLA Loneliness Scale—Version 3 [UCLA-LS; [26]. Secondary outcomes included the following: social isolation risk as measured by the lubben social network scale [LSNS; [27] measures of mental health included depression, using the patient health questionnaire-8 [PHQ-8; 28] social anxiety using the Mini-Social Phobia Inventory [MINI-SPIN; 29] quality of life using the European health interview survey—quality of life—8-item index [EUROHIS-QOL-8; 30] positive affect using the positive and negative affect schedule—SF [PANAS-SF; 31] and stress using the perceived stress scale-4 [PSS-4; 32] The following neighbourhood indicators were also measured as secondary outcomes: social cohesion and trust by Social Capital Scale [33] neighbourhood perception of change and importance [34]; and quality using the social relationship index—neighbourhood modified [35]. Bespoke measures to capture the presence of neighbourhood conflict, the number of neighbourhood contact, and a measure of COVID-19 social restrictions were also developed. See supplementary Table 1 for details of measures used in the published protocol [21] and Table 1 for internal consistencies.

### Statistical analysis

All analyses were conducted separately for each country to reflect differing responses to COVID-19 related social restrictions in place during the trials. In line with CONSORT reporting guidelines, balance at baseline between the intervention and waitlist control arms was established for all outcomes, and assessment of missingness for the primary outcome was conducted [36]. To assess intervention effects, intention-to-treat (ITT) analyses were used as it provides unbiased estimates of the impact of the intervention [37], comparing participants randomized to the KIND Challenge with those in the waitlist control. Analyses were conducted in MPlus 8.4.

For continuous outcome variables (e.g., loneliness, social isolation, depression, social anxiety, stress, quality of life, positive affect, and social cohesion and trust), linear regression models were conducted per outcome. For categorical outcome variables (neighbourhood stability, neighbourhood importance, social relationship index, neighbourhood conflict, number of neighbourhood contacts), bi-nominal and multi-nominal regression models were used per outcome. Explanatory co-variates were added (e.g., gender, age, baseline outcome), and the target variable intervention arm allocation (KIND Challenge vs. waitlist control) was added, with waitlist control as the reference group. Standardized beta coefficients indicate the magnitude of the intervention effect and Cohen’s *d* indicates the difference in the KIND Challenge and waitlist control post-test but taking into account pretest means and standard deviations of both groups [38].

A sensitivity analysis was conducted and is reported in the supplementary material (see supplementary data 5), to determine whether effects were robust and maintained once additional explanatory variables were controlled (e.g., loneliness, social isolation, depression, social anxiety, stress, quality of life, and positive affect, length of time as Nextdoor platform member, baseline neighbourhood social cohesion and trust, baseline number of neighbourhood contacts, COVID-19 related social restrictions).

## Results

Initial screening to check normality and score distribution (e.g., skew and kurtosis) found all scale outcome data to fall within acceptable levels for parametric analysis (skew ≤ 1) [39] but non-normality robust estimators (Mplus estimator MLR) were used to take into account slight deviation from non-normality in outcomes [40]. In line with CONSORT reporting standards, balance at baseline was established and confirmed randomization was successful (Table S2). A participant flow diagram is illustrated in Fig. 1. Predictors of missingness were identified (see supplementary data 3), and because missing cases were over 5% thresholds and missing at random, multiple imputation procedures were conducted using FIML in Mplus [41]. This enabled inclusion of both partially and completely observed cases in the analyses, reducing the bias associated with attrition. Table 3 shows descriptive data for outcomes at baseline and follow-up by intervention arm and country.

**Table 3:** Descriptive data for outcomes at baseline and follow-up by trial group and country

	USA				Australia				UK			
	Baseline		Follow-up		Baseline		Follow-up		Baseline		Follow-up	
	Challenge	Waitlist	Challenge	Waitlist	Challenge	Waitlist	Challenge	Waitlist	Challenge	Waitlist	Challenge	Waitlist
n	705	708	292	351	702	734	343	452	687	665	330	390
Loneliness	48.57 (12.34)	49.22 (11.69)	45.88 (12.31)	48.12 (12.06)	47.67 (12.00)	47.90 (12.18)	46.10 (12.93)	47.72 (12.55)	49.30 (12.37)	49.75 (12.08)	46.82 (12.09)	49.80 (12.72)
Social isolation	36.41 (15.47)	35.66 (15.35)	40.32 (16.13)	38.01 (16.14)	35.08 (15.00)	34.39 (15.09)	36.87 (15.67)	35.06 (15.81)	35.73 (15.55)	35.08 (16.46)	38.53 (15.43)	35.25 (15.89)
Depression	8.86 (6.20)	8.80 (5.96)	7.98 (6.22)	8.22 (6.00)	7.11 (5.79)	7.48 (6.19)	6.05 (5.66)	6.84 (5.96)	8.13 (6.37)	8.73 (6.60)	7.25 (6.35)	8.17 (6.61)
Social Anxiety	4.01 (3.42)	4.00 (3.44)	3.41 (3.32)	3.80 (3.43)	3.53 (3.29)	3.63 (3.28)	3.12 (3.13)	3.22 (3.12)	4.15 (6.35)	3.95 (3.51)	3.36 (3.24)	3.60 (3.43)
Quality of life	26.29 (7.08)	26.23 (6.89)	27.36 (7.57)	27.40 (7.11)	28.51 (6.70)	28.38 (6.84)	29.43 (6.89)	28.77 (6.87)	26.94 (7.13)	26.42 (7.08)	28.09 (7.03)	27.03 (7.31)
Stress	7.39 (3.67)	7.37 (3.47)	6.89 (3.63)	6.96 (3.58)	6.54 (3.44)	6.57 (3.44)	5.82 (3.54)	6.42 (3.48)	7.20 (3.40)	7.55 (3.51)	6.88 (3.43)	7.16 (3.68)
Positive affect	14.16 (4.48)	14.01 (4.62)	14.12 (4.32)	14.00 (4.62)	14.51 (4.33)	14.25 (4.65)	14.66 (4.62)	14.25 (4.64)	14.15 (4.89)	13.64 (4.79)	14.57 (4.94)	13.77 (4.90)
Social cohesion and trust	4.46 (2.09)	4.40 (2.08)	4.99 (2.02)	4.49 (2.09)	4.45 (2.09)	4.38 (2.09)	4.60 (2.18)	4.34 (2.12)	4.65 (2.09)	4.47 (2.16)	4.94 (2.07)	4.43 (2.22)
Neighbourhood stability %												
Stable	64.9%	66.4%	69.1%	67.5%	73.6%	76.9%	75.9%	73.8%	66.8%	66.6%	65.4%	68.1%
Improving	18.0%	18.1%	15.8%	14.9%	16.4%	14.7%	14.5%	14.1%	16.5%	15.0%	20.3%	14.0%
Declining	17.1%	15.5%	15.1%	17.6%	10.0%	8.4%	9.6%	12.1%	16.7%	18.4%	14.3%	17.9%
Neighbourhood importance												
Low	41.2%	42.8%	41.7%	43.8%	41.1%	41.3%	42.5%	45.5%	37.4%	34.9%	35.6%	38.8%
High	58.8%	57.2%	58.3%	56.2%	58.9%	58.7%	57.5%	54.6%	62.6%	65.1%	64.4%	61.2%
Supportive neighbour network (SRI) %												
Supportive	40.7%	39.5%	44.6%	39.5%	49.4%	47.2%	49.7%	46.5%	47.7%	44.6%	49.0%	42.5%
Aversive	11.2%	12.3%	8.3%	11.6%	10.4%	11.1%	7.2%	13.6%	9.4%	11.5%	8.0%	10.8%
Indifferent	6.8%	7.6%	5.4%	7.6%	11.3%	10.9%	9.3%	9.6%	5.1%	5.6%	6.1%	5.3%
Ambivalent	41.3%	40.5%	41.7%	41.3%	28.9%	30.5%	33.7%	30.2%	37.9%	38.3%	36.9%	41.4%
Neighbourhood conflict %												
Conflict	33.9%	35.3%	21.9%	30.4%	21.8%	22.8%	20.6%	19.8%	31.5%	34.6%	27.6%	34.5%
No conflict	66.1%	64.7%	78.1%	69.6%	78.2%	77.2%	79.4%	80.2%	68.5%	65.4%	72.4%	65.5%
No. of Neighbourhood contacts %												
0–5	56.7%	57.7%	47.8%	56.5%	49.9%	51.8%	48.5%	56.4%	46.4%	49.3%	44.4%	51.5%
6+	43.3%	42.3%	52.2%	43.5%	50.1%	48.2%	51.5%	43.6%	53.6%	50.7%	55.6%	48.5%

### Primary outcome

Findings showed significant intervention effects for reductions in loneliness post intervention in the USA (*B* = −0.06, SE = 0.02, *p* = 0.003, Cohen’s *d* = −0.13) and UK (*B* = -0.05, SE = 0.02, *p* = 0.026, Cohen’s *d* = −0.21), though the effect sizes are small (see Table S6). No such effects were observed in the Australia (*B* = −0.02, SE = 0.02, *p* = 0.331, Cohen’s *d* = −0.12). These findings were sensitive to the additional explanatory variables, highlighting the robustness of the trial effect in reducing loneliness (Table S7).

### Secondary outcomes

Significant intervention effects were found for social isolation in the USA. Those in the intervention group reported feeling less socially isolated after the four-week KIND challenge (*B* = 0.05, SE = 0.02, *p* = 0.033, Cohen’s *d* = 0.46); the effect size was moderate. Intervention effects were also seen for social anxiety, with lower social anxiety reported in the intervention groups relative to controls in the USA (*B* = −0.06, SE = 0.03, *p* = 0.027, Cohen’s *d* = −0.12) and Australia trial (*B* = −0.05, SE = 0.02, *p* = 0.031, Cohen’s *d* = −0.01). Significant intervention effects were found for reduced stress in Australia, as those in the intervention group reported lower levels of stress after the four-week KIND challenge (*B* = −0.05, SE = 0.02, *p* = 0.031, Cohen’s *d* = 0.17), the effect size was small. No intervention effects were observed for depression, quality of life, or positive affect (see supplementary data 5).

Effects of the intervention relative to the control group were seen for some of the neighbourhood variables. Post intervention, those in the intervention group, compared to control group, in the UK were more likely to report higher feelings of neighbourhood importance (*B* = 0.13, SE = 0.05, *p* = 0.039, OR = 1.62), and were twice as likely to report feeling in a stable, rather than declining, neighbourhood (*B* = 0.25, SE = 0.11, *p* = 0.022, OR = 1.97). Post intervention, those in the intervention group, compared to control group, in Australia were less likely to report feeling that they live in an aversive neighbourhood (*B* = −0.20, SE = 0.08, *p* = 0.011, OR = 0.46). Those in the intervention group, compared to control group, in the USA trial were more likely to report no issues of neighbourhood conflict (*B* = 0.09, SE = 0.05, *p* = 0.039, OR = 1.62) post intervention. Those in the intervention group, compared to control group, in the USA and Australia were more likely to know six or more neighbours post intervention (*B* = 0.09, SE = 0.05, *p* = 0.017, OR = 1.66, *B* = 0.08, SE = 0.04, *p* = 0.045, OR = 1.47); that was not the case in the UK. See Table S7 for full results.

*Sensitivity analysis.* After adjusting for additional explanatory factors, some of the mental health and neighbourhood outcomes changed in terms of reaching traditional significance levels. Overall, effect sizes were reduced, as is expected with more explanatory variables adding to the variance explained. The effects of the intervention trials in reducing loneliness in the USA and UK were maintained. A sensitivity analysis to follow-up the possibility of differential intervention effects found, the KIND Challenge did not have significant differential effects for those with lower feeling of loneliness at baseline. See supplementary data for overview (Tables S8 and S9). A summary of the intervention effects across loneliness, mental health, and neighbourhood relationship variables is summarized in Table 4.

**Table 4:** Summary of significant intervention effects per country trial

		Main analysis		
		USA	AUS	UK
Primary outcome	Loneliness	+ (+)		+ (+)
Mental health outcomes	Social isolation	+		
	Depression			
	Social anxiety	+ (+)		
	Quality of life			
	Stress		+	
	Positive affect			
Neighbourhood cohesion	Social cohesion and trust			
	Neighbourhood stability			+
	Social Relationship Index		+	
	Neighbourhood importance			+
	Neighbourhood conflict	+ (+)		
	No. of Neighbourhood contacts	+ (+)	+	

+ positive intervention effect; (+) positive intervention effect maintained in sensitivity analyses

*Feasibility, safety, compliance, acceptability ratings of the KIND Challenge*. The KIND challenge did not meet the feasibility criteria set of having a high retention rate. For those randomized into the KIND challenge, there was more than 40% drop out rate from the first baseline to post-intervention (see Fig. 1), however, the challenge was assessed to be safe as there were no unintended conflicts or adverse events reported across all countries. Compliance was assessed via dosage (i.e., number of kind acts completed), and on average, participants in the USA completed the most number of KIND acts at 3.01, followed by the UK at 2.78, and last Australia 2.58. Acceptability ratings were rated all above six out of a scale from one (not at all connected/meaningful/safe/positive/comfortable) to 10 (very connected/meaningful/safe/positive/comfortable). See Table S10 for details.

## Discussion

The health impacts of loneliness and social isolation have been extensively reported, but the evidence base to guide interventions in clinical and public health remains underdeveloped [3]. The current study involved parallel RCTs across three countries of a low-intensity and low/no-cost intervention using acts of kindness to address loneliness. Provision of support via acts of kindness to neighbours reduced loneliness, social isolation, and social anxiety and improved neighbourhood relationships over four weeks. Few interventions have been found to reduce loneliness and social isolation through rigorous RCT, and fewer have large sample sizes; thus, this large-scale international RCT contributes significantly to our understanding of effective interventions. It should be recognized that the effect sizes were relatively small, but this is consistent with the magnitude of effect of other trials of loneliness interventions [7]—despite these other interventions being far more resource-intensive and required trained professionals or volunteers. While it is not surprising that interventions such as the KIND Challenge did not demonstrate larger effects [42] given its context, in terms of implementation intensity (e.g., labor, effort, commitment, and cost), and its domain-specific meaning (e.g., the type of impact it is aiming to have) [42–44]. Nonetheless, even small effects may still potentially provide meaningful and clinically relevant change given evidence demonstrating a dose–response effect of a variety of indicators of social connection with mental health, health biomarkers (C-reactive protein, blood pressure, waist circumference, body mass index), and mortality [45, 46], such that every level of decrease in social disconnection was associated with subsequent decreases in risk. Taken together, our findings indicate that a simple low-intensity low-cost intervention to mobilize people to do acts of kindness, made immediate small reductions in loneliness, social isolation, social anxiety, stress, and made small improvements to neighbourhood relationships, even after controlling for known associates.

The effect of the intervention varied by outcome across countries. Loneliness was significantly reduced in the USA and UK, but not Australia. Reductions in social isolation were also demonstrated, but only in the USA. While other outcomes pertaining to neighbourhood relationships and engagement (i.e., conflict, attitudes, number of acquaintances) showed some improvements in all countries. The mixed findings may be accounted for by relative differences in compliance across the countries, resulting in a relatively higher average number of acts of kindness performed in the US. The compliance may be explained by familiarity with the Nextdoor platform, which is far more established in the USA, followed by the UK, with Australia, or due to country-related climate and politics. At the time of data collection, there were worldwide social restrictions in place to curb the spread of COVID-19, which differed greatly across Australia, the UK and USA. Indeed, Australia had mandated much stronger social restrictions compared to the UK and USA; thus, it is likely that this impacted the feasibility of engaging in the KIND Challenge.

These findings are consistent with approaches developed in positive psychology on promoting individual agency in acts of kindness that have shown improvements in happiness and psychological well-being for the actors involved [16, 47]. However, a recent review [48] yielded no clear evidence that acts of kindness can promote happiness, yet most studies reported in the review were limited to offers of financial support as the specified act of kindness. A strength of the current intervention was that participants chose their kindness activities meaning they selected activities that were feasible and meaningful to them, this autonomy may have enhanced the potential impact of kindness for our participants.

Our findings extend these benefits to loneliness and indicators of social connection, for which there is limited evidence. This offers an alternative direction in loneliness interventions, away from focusing on therapy and social support for affected individuals, towards encouraging more outward focused acts of kindness towards others to improve relationships quality. Providing support to others has been shown to be an important contributor to improved well-being in older adults, due to increased self-efficacy and feelings of usefulness to others [49]. There are considerable barriers to help seeking and receiving help that is responsive to needs; thus, self-initiated approaches may hold significant promise.

The current intervention was both accessible and safe. The intervention was flexible both in timing and the nature of the activity performed. Further, our study found that it is beneficial for reducing loneliness across age groups and levels of self-reported loneliness at baseline, with sensitivity analyses confirming that these factors did not alter the findings. Importantly, the intervention does not require any specific skill set or resources, making it scalable. The intervention was not delivered by health care or community practitioners; thus, it can be utilized in underserved and poorly resourced contexts, including rural communities and low-middle income countries.

It is noteworthy that the effects of the intervention were assessed for the individual providing the acts of kindness. There may be additional unmeasured benefits to those for whom the acts of kindness were directed. Given strong norms of reciprocity, such acts of kindness may be more likely to be reciprocated by others strengthening relationships over time. However, future research will need to examine potential mechanisms (e.g., reciprocity, purpose, belonging) leading to such benefits and whether benefits extend to others. This line of research could hold significant promise if such behaviors and subsequent reductions in loneliness spread through communities.

A significant contextual characteristic of this study is that it was conducted during the COVID-19 pandemic. The population-level impacts of the pandemic through increased stress, anxiety, depression, and social isolation are well documented [50–53]. Despite meta-analytic data demonstrating significant increases in both prevalence and loneliness globally during this time period [54], reductions in loneliness and improvement neighbourhood relationship indicators were achieved as a result of the intervention.

### Limitations

Despite the strengths of the KIND intervention, the effects were measured over four weeks; hence the longer-term durability of the outcomes observed remains unclear. Further, blinding the participants to the condition they were assigned was not possible due to the nature of the intervention. It is unclear how the effectiveness of the intervention may have been influenced the COVID-19 pandemic. While variability in social restrictions across and within countries were control for in our analyses, it is possible that the range and scope of acts of kindness may have differed had such restrictions not been in place. Similarly, another noted limitation is the relatively high representation of older adults and those (by virtue of using this platform) –who have an interest in connecting in the community. Notwithstanding these limitations, by conducting parallel RCTs in three countries we have been able to examine the consistency of intervention effects across social contexts. Each trial had sufficient power to detect differences in multiple outcomes of interest and validated and comprehensive measures were used that have been adopted widely in loneliness and isolation intervention trials. Noted attrition rates, with around 50% drop out from baseline to follow-up, may have been further compounded by the COVID-19 pandemic and related restrictions. While this is initially concerning, this is consistent with other survey-based research which has found similarly high attrition and non-completion rates in online questionnaires [55, 56] and online longitudinal online trials [57].

To mitigate this missing data analysis confirmed data was missing at random and multiple imputation could be used to maximize the dataset. Further strategies and guidance are needed how to reduce attrition or missingness for trials of this type in future. A further comment must acknowledge there is no correction for multiple testing across the secondary outcomes, methods available such as Bonferroni correction, tend to be extremely conservative and lead to high rate of false negatives [58] as such conclusion regarding the trial effects for should be taken with caution.

Consistent with many scientific studies of this nature, this study may inadvertently recruit participants that may inherently hold particular characteristics that may bias results. Furthermore, our study was conducted in the first six months of the pandemic which constrained us from recommending a higher dose of kindness (note. broader related constructs such as happiness is estimated at five acts of kindness per week) [59]. It is possible that we were not able to detect larger effects because we did not recommend a higher dose of kindness to participants.

## Conclusions

The onset of the COVID-19 pandemic has highlighted the importance of social connection and has served as a reminder of the importance of community [60]. While solutions to loneliness and social isolation have been widely implemented, these are rarely rigorously evaluated if evaluated at all [2]. The current study is the first study of its kind to evaluate the impact of an intervention focused on kindness to reduce loneliness in the general community. Given the prevalence of loneliness and social isolation globally [61], and the strength of clinical and public health evidence about the effects of these conditions [3], this intervention offers a promising approach that is scalable, and low cost, while effective in improving social, psychological, and neighbourhood factors.

## Supplementary Information

Below is the link to the electronic supplementary material.

Supplementary file1 (DOCX 78 KB)

## Data Availability

Data is available from the first author upon request.
