# Supplementary material for: The KIND Challenge community intervention to reduce loneliness and social isolation, improve mental health, and neighbourhood relationships: an international randomized controlled trial
Source: Soc Psychiatry Psychiatr Epidemiol. 2024 Aug 19;60(4):931–42. doi: 10.1007/s00127-024-02740-z (PMC12031860; doi:10.1007/s00127-024-02740-z)
Supplement: Supplementary file 1 — Supplementary file1 (DOCX 78 KB) [file 127_2024_2740_MOESM1_ESM.docx]

**The KIND Challenge community intervention to reduce loneliness and social isolation, improve mental health, and neighbourhood relationships: An international randomized controlled trial**

Michelle H Lim, PhD ^1 2^, Alexandra Hennessey, PhD^3^, Pamela Qualter, PhD^3^, Ben J Smith, PhD ^1^, Lily Thurston BA ^2^, Robert Eres, PhD ^2 4 5^, Julianne Holt-Lunstad, PhD ^2 6^

1. Prevention Research Collaboration, University of Sydney, Camperdown, NSW, Australia
2. Iverson Health Innovation Research Institute, Swinburne University of Technology, Hawthorn, Australia
3. Manchester Institute of Education, University of Manchester, Manchester, United Kingdom

Neurodisability and Rehabilitation, Murdoch Children’s Research Institute, Royal Children’s Hospital, Parkville, VIC, Australia

Department of Paediatrics, The University of Melbourne, Parkville, VIC, Australia

1. Department of Psychology, Neuroscience Center, Brigham Young University, Provo, Utah, United States of America

Corresponding Author:

Dr Michelle H Lim

University of Sydney

[michelle.h.lim@sydney.edu.au](mailto:michelle.h.lim@sydney.edu.au)

**Summary of online supplementary tables and additional data information**

- Table S1. Internal consistencies for measures used across 4 time points across Australia, USA, and UK
- Supplementary data 2: Balance at baseline
- Supplementary data 3: Predictors of missingness
- Supplementary data 4: Post hoc power calculations
- Supplementary data 5: Sensitivity analysis
- Table S6: Trial effects for loneliness and secondary outcomes of social isolation and mental health outcomes (standardised beta co-efficient)
- Table S7: Trial effects for neighborhood relationship quality and contact (standardised beta co-efficient)
- Table S8: Sensitivity analysis - Trial effects for loneliness and secondary outcomes of social isolation and mental health outcomes (standardised beta co-efficient)
- Table S9: Sensitivity analysis - Trial effects for neighborhood relationship quality and contact (standardised beta co-efficient)
- Table S10. Compliance and acceptability ratings across countries

Table S1. Internal consistencies for measures used across 4 time points across Australia, USA, and UK

| **Australia** | | | | | |
| --- | --- | --- | --- | --- | --- |
| **Measures** | **Time 1** | **Time 2** | **Time 3** | **Time 4** | **αs Range** |
| UCLA-LS | .947 | .956 | .956 | .960 | .95 - .96 |
| LSNS-18 |  |  |  |  |  |
| LSNS_Fa | .870 | .874 | .881 | .875 | .87 - .88 |
| LSNS_Ne | .885 | .896 | .882 | .896 | .88 - .90 |
| LSNS_Fr | .890 | .887 | .904 | .903 | .89 - .90 |
| SCS | .769 | .776 | .801 | .817 | .77 - .82 |
| PHQ-8 | .902 | .908 | .902 | .907 | .90 - .91 |
| Mini-SPIN | .862 | .858 | .872 | .866 | .86 - .87 |
| Positive Affect | .874 | .885 | .890 | .899 | .87 - .90 |
| PSS-4 | .779 | .754 | .773 | .797 | .75 - .80 |
| EUROHIS-QOL-8 | .898 | .901 | .905 | .913 | .90 - .91 |
| **USA** | | | | | |
| **Measure** | **Time 1** | **Time 2** | **Time 3** | **Time 4** | **αs Range** |
| UCLA-LS | .943 | .947 | .948 | .950 | .94 - .95 |
| LSNS-18 |  |  |  |  |  |
| LSNS_Fa | .859 | .863 | .877 | .878 | .86 - .88 |
| LSNS_Ne | .885 | .879 | .897 | .896 | .88 - .90 |
| LSNS_Fr | .887 | .905 | .887 | .893 | .89 - .91 |
| SCS | .772 | .773 | .784 | .793 | .77 - .79 |
| PHQ-8 | .885 | .892 | .903 | .898 | .89 - .90 |
| Mini-SPIN | .836 | .844 | .841 | .832 | .83 - .84 |
| Positive Affect | .845 | .860 | .848 | .868 | .85 - .87 |
| PSS-4 | .807 | .761 | .756 | .773 | .76 - .81 |
| EUROHIS-QOL-8 | .881 | .892 | .908 | .904 | .88 - .91 |
| **UK** | | | | | |
| **Measure** | **Time 1** | **Time 2** | **Time 3** | **Time 4** | **αs Range** |
| UCLA-LS | .945 | .953 | .951 | .954 | .94 - .95 |
| LSNS-18 |  |  |  |  |  |
| LSNS_Fa | .875 | .882 | .859 | .865 | .86 - .88 |
| LSNS_Ne | .880 | .885 | .886 | .883 | .88 - .89 |
| LSNS_Fr | .894 | .894 | .891 | .903 | .89 - .90 |
| SCS | .782 | .806 | .789 | .832 | .78 - .84 |
| PHQ-8 | .906 | .919 | .912 | .905 | .90 - .92 |
| Mini-SPIN | .870 | .863 | .858 | .859 | .86 - .87 |
| Positive Affect | .874 | .882 | .885 | .879 | .87 - .89 |
| PSS-4 | .765 | .754 | .748 | .747 | .75 - .77 |
| EUROHIS-QOL-8 | .890 | .898 | .892 | .899 | .89 - .90 |

*Note***.** UCLA-LS refers to University of California Loneliness Scale, LSNS refers to Lubben Social Network Scale, LSNS-FA refers to family subscale, LSNS-Ne refers to neighborhood subscale, LSNS-Fr refers to friend subscale, SCS refers to Social Capital Scale, PHQ-8 refers to Physical Health Questionnaire-8, Mini-SPIN refers to MINI- Social Phobia Inventory, PSS-4 refers to Perceived Stress Scale-4; EUROHIS-QOL-8 refers to European Health Interview Survey Quality of Life-8.

**Supplementary data 2: Balance at baseline**

### In the USA, a MANOVA found no main effect of intervention arm at baseline across the primary outcome loneliness and secondary mental health outcomes (USA: *F* (7, 1389) = 0.44, *p* = .877). A series of t-tests found no significant group differences at baseline for neighborhood outcome social cohesion and trust (USA: t = -0.58, df = 1389, p = .563), neighborhood stability (USA: *X*^2^ = 0.71, df = 2, *p* = .701), neighborhood importance (USA: *X*^2^ = 0.40, df = 1, *p* = .528), supportive neighbour network (USA: *X*^2^ = 0.89, df = 3, p = .827) neighborhood conflict (USA: *X*^2^ = 0.29, df = 1, *p* = .589) or number of known neighbors (USA: *X*^2^ = 0.15, df = 1, *p* = .702).

In the UK, a MANOVA found a main effect of intervention arm across the primary outcome loneliness and secondary mental health outcomes (UK: *F* (7, 1307) = 2.21, *p* = .031), with a significant difference at baseline for stress only (UK: *F*(1, 1313) = 4.57, *p* = .033), stress was significantly higher in the intervention arm. A series of t-tests found no intervention arms differences at baseline on neighborhood outcomes social cohesion and trust (UK: *t* = -1.56, *df* = 1356, *p* = .118), neighborhood stability (UK: *X*^2^ = 1.07, *df* = 2, *p* = .585), neighborhood importance (UK: *X*^2^ = 0.91, *df* = 1, *p* = .341), social relationship index (UK: *X*^2^ = 2.29, *df* = 3, *p* = .515), neighborhood conflict (UK: *X*^2^ = 1.46, *df* = 1, *p* = .227), or number of known neighbors (UK: *X*^2^ = 1.19, *df* = 1, *p* = .276).

### In Australia, a MANOVA found no significant main effect of intervention arm at baseline across the primary outcome loneliness and secondary mental health outcomes (AUS: *F* (7, 1411) = 0.59, *p* = .763). A series of *t*-tests found no significant group differences at baseline for neighborhood outcomes; social cohesion and trust (AUS: *t* = -0.57, *df* = 1426, *p* = .571), neighborhood stability (AUS: *X*^2^ = 2.17, *df* = 2, *p* = .337), neighborhood importance (AUS: *X*^2^ = 0.04, *df* = 1, *p* = .948), social relationship index (AUS: *X*^2^ = 0.95, *df* = 3, *p* = .815) neighborhood conflict (AUS: *X*^2^ = 0.22, *df* = 1, *p* = .643), or number of known neighbors (AUS: *X*^2^ = 0.56, *df* = 1, *p* = .454).

In conclusion, balance at baseline was established, and conforms randomization was successful.

**Supplementary data 3: Predictors of missingness**

Analysis of data for primary outcome, loneliness, post four-week intervention revealed that in the in the USA trial 54.8% of cases were missing at follow-up, in the UK it was 49.1% and Australia, 45.2% of cases were missing at follow-up. The remaining cases were complete. Logistic regression identified a range of variables that predicted partially observed status (e.g., missingness) ^45^. Missing data were more likely to be from participants assigned to the Challenge condition (USA: B = 0.34, SE = 0.11, *p* = .002; UK B = 0.42, SE = 0.12, *p* < .001; AUS: B = 0.50, SE = 0.11, *p* < .001), younger participants (UK: B = -0.02, SE = 0.00, *p* < .001; AUS: B = -0.02, SE = 0.00, *p* < .001), and those scoring lower on social cohesion and trust (USA: B = -0.10, SE = 0.04, *p* = .008; AUS: B = -0.08, SE = 0.04, *p* = .023); in the UK, men and those who classified themselves as living in an indifferent neighborhood network compared to supportive network were more likely to have missing data (UK: B = 0.38, SE = 0.14, *p* = .005, B = -0.57, SE = 0.29, *p* = .049). In the USA, missingness was predicted by higher loneliness, greater social isolation, lower positive affect, and fewer instances of neighborhood conflict (USA: B = -0.02, SE = 0.01, *p* = .014; B = -0.01, SE = 0.01, *p* = .027, B = 0.04, SE = 0.02, *p* = .012, B = .34, SE = 0.13, *p* = .010). Data was confirmed as missing at random (e.g., conditional on observed data) as opposed to missing completely as random. ^46^

**Supplementary data 4: Post hoc power calculations**

Power calculations were calculated using PowerUp!^[[1]](#footnote-1)^. Power was set at 0.80 and an Alpha threshold of 0.05. Pre-post correlations for loneliness in the current study were USA *r* = .85, UK *r* = .87, and Australia *r* = .88 (pre-test loneliness was explaining up to 77% of the variance in post-test loneliness). Therefore, for respective sample sizes of USA *n*=1,410, UK *n* = 1,412, and Australia *n* = 1,452 participants per country and with up to 14 co-variates for the primary outcomes of loneliness, a minimum detectable effect size (MDES) for a trial effect was USA = .08, UK = .07 and Australia = .07.

**Supplementary data 5: Sensitivity analysis**

Sensitivity analysis controlling for additional co-variates found some noteworthy changes in intervention effects. Mental health related variables at baseline were controlled for (e.g., depression, social anxiety, stress, quality of life, and positive affect) given their known associations with the response variable loneliness. Additionally, variables related to community connection at baseline were controlled for (e.g., length of time as Nextdoor platform member, baseline neighbourhood social cohesion and trust, baseline number of neighbourhood contact) given the potential for these factors to affect the response variable loneliness. Finally, controlling for COVID-19 levels of social restriction was needed to account for differences in regional social restrictions that would impact on ability to engage and interact with neighbours and the community.

The intervention effects for the primary outcome, loneliness, were maintained, but this was not the case for all secondary outcomes. Considering mental health secondary outcomes, the lower levels of social anxiety reported in the intervention group in USA was maintained (*B* = -0.06, SE = 0.03, *p* = .022), but the effect for feelings of being less socially isolated in the USA was non-significant (B = 0.04, SE = 0.02, *p* = .059) as was the effect for reduced stress in Australia (B = -0.04, SE = 0.02, *p* = .074) (See Table S6 below). The KIND Challenge did not have differential effects for those with lower levels of loneliness at baseline (USA: *B* = -0.13, SE = 0.09, *p* = .131, UK: UK: *B* = -0.14, SE = 0.08, *p* = .091, Australia: *B* = 0.05, SE = 0.07, *p* = .441).

Similarly, following the sensitivity analysis, there were slight changes in some of neighborhood relationships findings. For example, the significant intervention effects for the neighborhood variables were maintained in the USA; they were more likely to report no issues of neighborhood conflict; *B* = 0.11, SE = 0.05, *p* = .023, OR = 1.62), and participants were still more likely to know six or more neighbors post intervention (*B* = 0.09, SE = 0.05, *p* = .042, OR = 1.61). Australians reported feeling less likely that they live in an aversive neighborhood were maintained (*B* = -0.18, SE = 0.08, *p* = .023, OR = 0.47), but being more likely to know six or more neighbors post intervention was no longer significant (*B* = 0.05, SE = 0.04, *p* = .164, OR = 1.30). In the UK, both the effects on increased feelings of neighborhood importance and feeling in a stable rather than declining neighborhood fell below significance thresholds (*B* = 0.02, SE = 0.04, *p* = .627, OR = 1.11, *B* = 0.15, SE = 0.09, *p* = .102, OR = 1.70) (See Table S7 below).

Table S6: Trial effects for loneliness and secondary outcomes of social isolation and mental health outcomes (standardised beta co-efficient)

|  | | Loneliness | Social isolation | Depression | Social anxiety | Quality of life | Stress | Positive affect |
| --- | --- | --- | --- | --- | --- | --- | --- | --- |
|  | | **USA** | | | | | | |
| Trial if KIND Challenge | | -0.05 (0.02)* | 0.05 (0.02)* | -0.01 (0.02) | -0.06 (0.03)* | -0.01 (0.02) | -0.01 (0.03) | 0.02 (0.03) |
| Age mean | | 0.01 (0.02) | -0.02 (0.02) | -0.01 (0.02) | -0.02 (.03) | .01 (0.02) | -0.02 (0.03) | 0.03 (0.03) |
| Gender (compared to female) | |  |  |  |  |  |  |  |
|  | If male | 0.00 (0.02) | 0.01 (0.02) | -0.01 (0.02) | 0.01 (0.03) | 0.01 (0.02) | 0.03 (0.03) | -0.02 (0.03) |
|  | If other | 0.00 (0.02) | -0.02 (0.02) | -0.02 (0.04) | 0.01 (0.03) | 0.01 (0.02) | -0.02 (0.03) | -0.04 (0.03) |
| Respective baseline outcome score | | 0.85 (0.01)** | 0.85 (0.01)** | 0.81 (0.02)** | 0.76 (0.02)** | 0.87 (.01)** | 0.75 (0.02)** | 0.71 (0.02)** |
|  | |  |  |  |  |  |  |  |
|  | | **AUS** | | | | | | |
| Trial if KIND Challenge | | -0.02 (0.02) | -0.01 (0.02) | -0.02 (0.02) | 0.03 (0.02) | 0.02 (0.02) | -0.05 (0.02)* | 0.01 (0.02) |
| Age | | -0.00 (0.02) | 0.05 (0.02) | -0.03 (0.02) | 0.00 (0.02) | 0.00 (0.02) | -0.04 (0.03) | 0.06 (0.03)* |
| Gender (compared to female) | |  |  |  |  |  |  |  |
|  | If male | -0.01 (0.02) | 0.02 (0.02) | 0.03 (0.02)† | 0.02 (0.02) | -0.01 (0.02) | -0.00 (0.03) | -0.01 (0.02) |
|  | If other | -0.03 (0.01)* | -0.02 (0.01)* | 0.02 (0.02) | 0.01 (0.02) | -0.02 (0.01)* | -0.01 (0.01) | 0.02 (0.03) |
| Respective baseline outcome score | | 0.88 (0.01)** | 0.88 (0.01)** | 0.82 (0.02)** | 0.79 (0.02)** | 0.89 (0.01)** | 0.73 (0.02)** | 0.76 (0.02)** |
|  | | **UK** | | | | | | |
| Trial if KIND Challenge | | -0.06 (0.02)* | 0.03 (0.02) | 0.00 (0.02) | -0.03 (0.02) | 0.02 (0.02) | 0.02 (0.03) | 0.01 (0.03) |
| Age | | -0.01 (0.02) | -0.01 (0.02) | -0.01 (0.02) | -0.06 (0.02)** | 0.02 (0.02) | -0.06 (0.03)* | -0.02 (0.03) |
| Gender (compared to female) | |  |  |  |  |  |  |  |
|  | If male | -0.01 (0.02) | -0.02 (0.02) | -0.00 (0.02) | -0.00 (0.03) | 0.02 (0.02) | 0.00 (0.03) | -0.01 (0.02) |
|  | If other | 0.01 (0.03) | -0.00 (0.02) | 0.02 (0.02) | -0.00 (0.01) | -0.00 (0.02) | 0.01 (0.02) | -0.03 (0.01)* |
| Respective baseline outcome score | | 0.87 (0.01)** | 0.88 (0.01)** | 0.83 (0.02)** | 0.76 (0.2)** | 0.88 (0.01)** | 0.71 (0.02)** | 0.75 (0.02)** |

†sign .10, *sign .05, **sign .001

Table S7: Trial effects for neighborhood relationship quality and contact (standardised beta co-efficient)

|  | | | Social cohesion and trust | Neighborhood stability (reference group declining) | | Social Relationship Index  (reference group supportive) | | | Neighborhood importance (reference group low) | Neighborhood conflict (reference group at least one instance of conflict) | No. of Neighborhood contacts (reference group <5 contacts) |
| --- | --- | --- | --- | --- | --- | --- | --- | --- | --- | --- | --- |
|  | | |  | ***If stable*** | ***If improving*** | ***If ambivalent*** | ***If aversive*** | ***If indifferent*** | ***If high*** | ***If no conflict*** | ***If 6+ contacts*** |
|  | | | **USA** | | | | | | | | |
| Trial if KIND Challenge | | | 0.04 (0.03) | 0.04 (0.012) | 0.05 (0.11) | -0.05 (0.06) | -0.10 (0.07) | -0.11 (0.10) | 0.01 (0.06) | 0.12 (0.05)* | 0.09 (0.05)* |
| Age mean | | | -0.010 (0.02) | -0.02 (0.10) | -0.02 (0.012) | -0.10 (0.06) | 0.03 (0.08) | -0.23 (0.09)* | -0.00 (0.06) | 0.05 (005) | 0.07 (0.05) |
| Gender (compared to female) | | | 0.77 (0.02)** | 0.78 (1.27)  (If stable)  1.17 (1.88)  (If improving) | 1.27 (0.06)** (If stable)  1.05 (0.13)**  (If improving) | 0.48 (0.10)**  (If aversive)  -0.02 (0.09)  (If indifferent)  0.57 (0.08)**  (If ambivalent) | 0.74 (0.07)**  (If aversive)  0.24 (0.08)*  (If indifferent)  0.49 (0.09)**  (If ambivalent) | 0.45 (0.10)**  (If aversive)  0.03 (0.07)**  (If indifferent)  -0.04 (0.14)  (If ambivalent) | 0.44 (0.04)**  (If high) | 0.44 (.04)**  (If no conflict) | 0.68 (0.03)**  (If 6+ contacts) |
|  | | If male | -0.10 (.03)** | -0.05 (0.13) | -0.13 (0.11) | 0.07 (0.06) | 0.14 (0.07)* | -0.12 (0.14) | 0.02 (0.05) | -0.10 (0.04)* | 0.03 (0.05) |
|  | | If other | -0.03 (0.02) | -0.45 (2.79) | 0.05 (0.12) | 0.73 (0.08) | 0.62 (0.07)** | 0.72 (0.07)** | -0.38 (0.05)** | -0.03 (0.04) | -0.05 (0.03) |
| Respective baseline outcome score | | |  |  |  |  |  |  |  |  |  |
|  | | | **AUS** | | | | | | | | |
| Trial if KIND Challenge | | | 0.01 (0.02) | 0.04 (0.013) | 0.10 (0.45) | 0.03 (0.07) | -0.20 (0.08)* | -0.02 (0.09) | 0.05 (0.04) | -0.00 (0.04) | 0.08 (0.04)* |
| Age mean | | | 0.03 (0.03) | 0.16 (0.13) | 0.20 (0.86)† | 0.06 (0.07) | -0.06 (0.08) | -0.13 (0.16) | 0.15 (0.04)** | 0.01 (0.05) | -0.00 (0.04) |
| Gender (compared to female) | | |  |  |  |  |  |  |  |  |  |
|  | If male | | -0.01 (0.02) | 0.09 (0.13) | 0.05 (0.26) | 0.16 (0.07)* | 0.07 (0.08) | -0.04 (0.10) | -0.04 (0.04) | 0.08 (0.05) | -0.00 (0.04) |
|  | If other | | 0.02 (0.03) | -0.21 (0.05)** | 0.63 (4.02) | -0.01 (0.08) | 0.07 (0.06) | -0.33 (2.78) | 0.02 (0.02) | 0.02 (0.05) | -0.28 (0.06)** |
| Respective baseline outcome score | | | 0.78 (0.02)** | 0.85 (0.18)** (If stable)  1.41 (0.07)**  (If improving) | 1.06 (4.47)  (If stable)  0.98 (4.12)  If improving) | 0.83 (0.07)**  (If aversive)  0.09 (0.10)  (If indifferent)  0.81 (0.08)**  (If ambivalent) | 0.94 (0.04)**  (If aversive)  0.48 (0.06)**  (If indifferent)  0.48 (0.08)**  (If ambivalent) | 0.81 (0.77)  (If aversive)  0.65 (0.62)  (If indifferent)  0.24 (0.26)  (If ambivalent) | 0.58 (0.03)**  (If high) | 0.44 (0.03)**  (If no conflict) | 0.60 (0.03)**  (If 6+ contacts) |
|  | | | **UK** | | | | | | | | |
| Trial if KIND Challenge | | | 0.04 (0.02)† | 0.25 (0.11)* | 0.08 (0.10) | -0.08 (0.08) | -0.07 (0.08) | 0.02 (0.12) | 0.13 (0.05)* | 0.07 (0.05) | 0.01 (0.04) |
| Age mean | | | 0.02 (0.03) | -0.10 (0.12) | -0.06 (0.10) | -0.02 (0.08) | 0.02 (0.09) | -0.16 (0.50) | -0.03 (.05) | 0.01 (0.05) | 0.09 (0.04)* |
| Gender (compared to female) | | |  |  |  |  |  |  |  |  |  |
|  | If male | | 0.02 (0.03) | 0.06 (0.12) | 0.05 (0.10) | -0.05 (0.08) | -0.07 (0.08) | -0.26 (0.22) | 0.02 (0.04) | 0.01 (0.05) | 0.02 (0.05) |
|  | If other | | 0.02 (0.01)† | 0.02 (1.27) | 0.44 (0.68) | 0.06 (0.09) | -0.31 (0.54) | -0.43 (1.51) | -0.24 (0.04)** | -0.03 (.08) | -0.04 (0.04) |
| Respective baseline outcome score | | | 0.80 (0.02)** | 1.01 (0.10)** (If stable)  1.14 (0.08)**  (If improving) | 1.12 (0.42)** (If stable)  0.77 (0.31)*  (If improving | 0.73 (0.11)**  (If aversive)  0.22 (0.08)*  (If indifferent)  0.93 (0.08)**  (If ambivalent) | 0.94 (0.13)**  (If aversive)  0.29 (0.08)**  (If indifferent)  0.65 (0.12)**  (If ambivalent) | 0.76 (0.50)  (If aversive)  0.52 (0.34)  (If indifferent)  0.02 (0.21)  (If ambivalent) | 0.33 (0.04)**  (If high) | 0.51 (0.03)**  (If no conflict) | 0.70 (0.02)**  (If 6+ contacts) |

†sign .10, *sign .05, **sign .001

Table S8: Sensitivity analysis - Trial effects for loneliness and secondary outcomes of social isolation and mental health outcomes (standardised beta co-efficient)

|  | | | | Loneliness | Social isolation | Depression | Social anxiety | Quality of life | Stress | Positive affect |
| --- | --- | --- | --- | --- | --- | --- | --- | --- | --- | --- |
|  | | | | **USA** | | | | | | |
| Trial if KIND Challenge | | | | -0.05 (0.02)* | 0.04 (0.02)† | -0.00 (0.02) | -0.06 (0.03)* | -0.01 (0.02) | -0.00 (0.03) | 0.01 (0.03) |
| Age | | | | 0.02 (0.02) | -0.03 (0.02) | -0.01 (0.02) | -0.03 (0.03) | 0.01 (0.02) | 0.03 (0.03) | 0.02 (0.03) |
| Gender (compared to female) | | | |  |  |  |  |  |  |  |
|  | | | If male | 0.00 (0.02) | 0.01 (0.02) | -0.02 (0.02) | -0.01 (0.03) | 0.01 (0.02) | 0.01 (0.02) | 0.01 (0.03) |
|  | | | If other | 0.00 (0.02) | -0.02 (0.02) | -0.04 (0.04) | 0.00 (0.03) | 0.01 (0.02) | -0.03 (0.02) | -0.01 (0.02) |
| Length of time in Nextdoor (compared to <6 months) | | | |  |  |  |  |  |  |  |
|  | | | If 1 year | -0.02 (0.02) | -0.04 (0.02)† | -0.02 (0.03) | 0.02 (0.03) | -0.01 (0.02) | 0.04 (0.03) | 0.00 (0.03) |
|  | | | If over year | -0.00 (0.02) | -0.02 (0.03) | 0.01 (0.03) | -0.03 (0.03) | -0.02 (0.02) | 0.05 (0.03)† | 0.00 (0.04) |
| Baseline loneliness | | | | 0.70 (0.03)** | -0.06 (0.04)† | 0.17 (0.04)** | 0.12 (0.05)* | -0.07 (0.03)† | 0.18 (0.40)** | -0.18 (0.05)** |
| Baseline social isolation | | | | -0.12 (0.04)** | 0.78 (0.03)** | 0.04 (0.04) | -0.02 (0.04) | 0.04 (0.03) | -0.01 (0.04) | 0.05 (0.04) |
| Baseline depression | | | | 0.09 (0.03)** | -0.02 (0.03) | 0.72 (0.03)** | 0.11 (0.4)* | -0.05 (0.03)† | 0.16 (0.04)** | -0.07 (0.04)† |
| Baseline social anxiety | | | | 0.05 (0.03)† | -0.01 (0.03) | 0.02 (0.03) | 0.66 (0.03)** | -0.01 (0.03) | 0.02 (0.03) | 0.01 (0.03) |
| Baseline outcome | | | | - | - | - | - | 0.78 (0.03)** | 0.52 (0.04)** | 0.56 (0.03)** |
| Baseline Neighborhood social cohesion and trust | | | | -0.02 (0.03) | 0.04 (0.03) | -0.00 (0.03) | 0.07 (0.03)* | -0.03 (0.02) | -0.00 (0.03) | -0.03 (0.03) |
| Baseline number of Neighborhood contacts (if 6+) | | | | 0.05 (0.02)* | -0.00 (0.03) | -0.01 (0.03) | 0.05 (0.03) | -0.02 (0.02) | 0.03 (0.03) | -0.01 (0.03) |
| COVID-19 related perceived restrictions | | | | 0.02 (0.02) | -0.01 (0.02) | 0.02 (0.03) | -0.00 (0.03) | -0.04 (0.02)† | 0.01 (0.03) | -0.01 (0.03) |
|  | | | | **Aus** | | | | | | |
| Trial if KIND Challenge | | | | -0.01 (0.02) | -0.00 (0.02) | -0.01 (0.02) | 0.03 (0.02) | 0.01 (0.02) | -0.04 (0.02)† | 0.00 (0.02) |
| Age | | | | 0.02 (0.02) | 0.03 (0.02) | -0.02 (0.02) | 0.01 (0.03) | -0.01 (0.02) | -0.02 (0.03) | 0.03 (0.03) |
| Gender (compared to female) | | | |  |  |  |  |  |  |  |
|  | If male | | | -0.00 (0.02) | 0.01 (0.02) | 0.02 (0.02) | 0.01 (0.02) | -0.00 (0.02) | -0.00 (0.02) | -0.00 (0.02) |
|  | If other | | | -0.02 (0.02) | -0.01 (0.01)* | 0.02 (0.03) | 0.01 (0.02) | -0.02 (0.01)* | -0.01 (0.01) | 0.03 (0.02) |
| Length of time in Nextdoor (compared to <6 months) | | | |  |  |  |  |  |  |  |
|  | | If 1 year | | 0.03 (0.02) | -0.03 (0.02) | -0.01 (0.02) | -0.02 (0.03) | 0.00 (0.02) | -0.05 (0.03)† | 0.00 (0.03) |
|  | | If over year | | 0.01 (0.02) | -0.02 (0.02) | -0.01 (0.02) | 0.02 (0.02) | 0.01 (0.02) | -0.05 (0.03)† | 0.02 (0.02) |
| Baseline loneliness | | | | 0.70 (0.03)** | -0.10 (0.03)** | 0.05 (0.04) | -0.02 (0.04) | -0.01 (0.03) | 0.12 (0.05)* | -0.09 (0.04)* |
| Baseline social isolation | | | | -0.11 (0.03)** | 0.76 (0.03)** | -0.07 (0.03)* | -0.09 (0.04)* | 0.06 (0.03)* | -0.02 (0.04) | 0.05 (0.04) |
| Baseline depression | | | | 0.08 (0.03)** | -0.02 (0.03) | 0.71 (0.03)** | 0.13 (0.04)** | -0.06 (0.03)* | 0.17 (0.04)** | -0.11 (0.04)* |
| Baseline social anxiety | | | | 0.08 (0.02)** | -0.01 (0.02) | 0.11 (0.03)** | 0.70 (0.03)** | -0.01 (0.02) | 0.03 (0.03) | 0.01 (0.03) |
| Baseline outcome | | | | - | - | - | - | 0.79 (0.03)** | 0.52 (0.04)** | 0.63 (0.03)** |
| Baseline Neighborhood social cohesion and trust | | | | -0.04 (0.02) | 0.04 (0.02) | 0.03 (0.03) | 0.01 (0.03) | 0.03 (0.02) | -0.02 (0.03) | 0.00 (0.03) |
| Baseline number of Neighborhood contacts (if 6+) | | | | 0.02 (0.02)† | 0.02 (0.02) | 0.01 (0.03) | 0.03 (0.03) | -0.01 (0.02) | 0.05 (0.03)† | -0.00 (0.03) |
| COVID-19 related perceived restrictions | | | | -0.01 (0.02) | -0.02 (0.02) | -0.01 (0.02) | 0.02 (0.02) | -0.02 (0.02) | -0.01 (0.02) | 0.03 (0.02) |
|  | | | | **UK** | | | | | | |
| Trial if KIND Challenge | | | | -0.05 (0.02)* | 0.02 (.02) | 0.00 (0.02) | -0.02 (0.02) | 0.01 (0.02) | 0.03 (0.03) | 0.00 (0.03) |
| Age | | | | 0.00 (0.02) | -0.03 (0.02) | -0.01 (0.02) | -0.05 (0.03) | 0.01 (0.02) | -0.05 (0.03) | -0.06 (0.03)* |
| Gender (compared to female) | | | |  |  |  |  |  |  |  |
|  | If male | | | -0.02 (0.02) | -0.02 (0.02) | -0.01 (0.02) | -0.02 (0.03) | 0.02 (0.02) | -0.02 (0.03) | 0.01 (0.02) |
|  | If other | | | 0.01 (0.02) | -0.00 (0.02) | 0.02 (0.02) | -0.01 (0.01) | -0.00 (0.02) | 0.00 (0.02) | -0.02 (0.01)† |
| Length of time in Nextdoor (compared to <6 months) | | | |  |  |  |  |  |  |  |
|  | | If 1 year | | 0.02 (0.02) | -0.02 (.02) | -0.03 (0.02) | -0.01 (0.03) | -0.02 (0.02) | 0.02 (0.03) | 0.03 (0.03) |
|  | | If over year | | 0.02 (0.02) | 0.00 (0.02) | -0.00 (0.02) | -0.06 (0.03)* | -0.02 (0.02) | -0.02 (0.03) | 0.01 (0.03) |
| Baseline loneliness | | | | 0.072 (0.03)** | -0.12 (0.04)** | 0.06 (0.04)† | 0.09 (0.04)* | 0.01 (0.03) | 0.07 (0.05) | -0.01 (0.05) |
| Baseline social isolation | | | | -0.12 (0.03)** | 0.76 (0.03)** | -0.02 (0.04) | 0.04 (0.04) | 0.04 (0.04) | -0.03 (0.05) | 0.07 (0.05) |
| Baseline depression | | | | 0.06 (0.03)* | -0.04 (0.03) | 0.77 (0.03)** | 0.11 (0.04)* | -0.13 (0.04)** | 0.25 (0.04)** | -0.14 (0.04)** |
| Baseline social anxiety | | | | 0.03 (0.03) | 0.02 (0.03) | 0.01 (0.03) | 0.66 (0.03)** | 0.01 (0.03) | -0.03 (0.03) | 0.04 (0.04) |
| Baseline outcome | | | | - | - | - | - | 0.75 (0.03)** | 0.49 (0.04)** | 0.62 (0.03)** |
| Baseline Neighborhood social cohesion and trust | | | | -0.03 (0.02) | -0.02 (0.02) | -0.01 (0.03) | -0.04 (0.03) | 0.03 (0.03) | -0.03 (0.03) | 0.07 (0.03)* |
| Baseline number of Neighborhood contacts (if 6+) | | | | -0.00 (0.02) | 0.03 (0.02) | 0.01 (0.03) | 0.03 (0.03) | 0.03 (0.02) | 0.01 (0.03) | -0.00 (0.03) |
| COVID-19 related perceived restrictions | | | | -0.01 (0.02) | -0.00 (0.02) | 0.04 (0.02) | 0.01 (0.03) | 0.00 (0.07) | 0.04 (0.03) | 0.04 (0.03) |

†sign .10, *sign .05, **sign .001

Table S9: Sensitivity analysis - Trial effects for neighborhood relationship quality and contact (standardised beta co-efficient)

|  | | | Social cohesion and trust | Neighborhood stability (reference group declining) | | Social Relationship Index  (reference group supportive) | | | Neighborhood importance (reference group low) | Neighborhood conflict (reference group at least one instance of conflict) | No. of Neighborhood contacts (reference group <5 contacts) |
| --- | --- | --- | --- | --- | --- | --- | --- | --- | --- | --- | --- |
|  | | |  | ***If stable*** | ***If improving*** | ***If ambivalent*** | ***If aversive*** | ***If indifferent*** | ***If high*** | ***If no conflict*** | ***If 6+ contacts*** |
|  | | | **USA** | | | | | | | | |
| Trial if KIND Challenge | | | 0.04 (0.03) | 0.02 (0.09) | 0.02 (0.10) | -0.04 (0.06) | -0.06 (.07) | -0.14 (0.10) | 0.02 (0.04) | 0.11 (0.05)* | 0.09 (0.05)* |
| Age | | | -0.01 (0.03) | -0.02 (0.10) | 0.04 (0.11) | -0.06 (0.07) | 0.02 (0.08) | -0.21 (0.11) | 0.07 (0.05) | 0.06 (0.05) | 0.09 (0.05) |
| Gender (compared to female) | | |  |  |  |  |  |  |  |  |  |
|  | | If male | -0.09 (0.03)** | -0.02 (0.09) | -0.08 (0.09) | 0.06 (0.06) | 0.11 (0.06)† | -0.11 (0.13) | -0.06 (0.04) | -0.10 (0.04)* | 0.04 (0.05) |
|  | | If other | -0.02 (0.02) | -0.31 (2.74) | 0.12 (0.12) | 0.71 (0.06)** | 0.45 (0.07)** | 0.62 (0.06)** | 0.01 (0.02) | -0.02 (0.05) | -0.2 (0.03) |
| Length of time in Nextdoor (compared to <6 months) | | |  |  |  |  |  |  |  |  |  |
|  | | If 1 year | 0.01 (0.03) | 0.13 (0.18) | 0.14 (0.12) | 0.05 (0.08) | -0.12 (0.08) | -0.16 (0.14) | 0.03 (0.05) | -0.02 (0.06) | 0.13 (0.05)* |
|  | | If over year | 0.01 (0.03) | 0.02 (0.11) | -0.07 (0.12) | -0.04 (.08) | -0.05 (0.08) | 0.06 (0.12) | -0.07 (0.05) | -0.04 (0.06) | 0.09 (0.06) |
| Baseline loneliness | | | -0.04 (0.05) | -0.39 (0.49) | -0.31 (0.18)† | 0.09 (0.11) | 0.19 (0.11) | 0.02 (0.17) | 0.11 (0.08) | -0.04 (0.09) | 0.09 (0.07) |
| Baseline social isolation | | | 0.12 (0.05)* | -0.07 (0.16) | 0.08 (0.16) | -0.08 (0.10) | -0.31 (0.11) | -0.32 (0.16) | 0.07 (0.07) | -0.03 (0.08) | 0.12 (0.07)† |
| Baseline depression | | | -0.02 (0.03) | 0.08 (0.15) | 0.10 (0.12) | -0.04 (0.09) | -0.05 (0.09) | -0.07 (0.12) | 0.04 (0.06) | -0.12 (0.06)† | -0.12 (0.06)* |
| Baseline social anxiety | | | 0.03 (0.03) | 0.16 (0.21) | 0.18 (0.11) | 0.08 (0.08) | -0.11 (0.08) | 0.17 (0.11) | -0.03 (0.05) | 0.08 (0.06) | 0.07 (0.06) |
| Baseline Neighborhood social cohesion and trust | | | 0.70 (0.03)** | 0.19 (0.23) | 0.23 (0.11)* | -0.21 (0.09) | -0.41 (0.10)** | -0.20 (0.14) | 0.18 (0.05)** | 0.05 (0.06) | 0.06 (0.05) |
| Baseline number of Neighborhood contacts (if 6+) | | | 0.02 (0.03) | 0.10 (0.16) | -0.10 (0.12) | 0.07 (0.15) | -0.06 (0.17) | -0.16 (0.27) | 0.14 (0.05)* | -0.06 (0.05) | 0.61 (0.04)** |
| COVID-19 related perceived restrictions | | | -0.02 (0.03) | -0.06 (0.12) | -0.16 (0.10) | -0.04 (0.07) | 0.13 (0.07)† | -0.12 (0.11) | 0.01 (0.05) | 0.07 (0.05) | -0.04 (0.05) |
| Respective baseline outcome score | | | 0.77 (0.02)** | 0.63 (0.71)  (If stable)  0.96 (1.06)  (If improving) | 1.01 (0.09)** (If stable)  0.80 (0.13)**  (If improving) | 0.35 (0.10)**  (If aversive)  -0.07 (0.10)  (If indifferent)  0.57 (0.07)**  (If ambivalent) | 0.44 (0.07)**  (If aversive)  0.10 (0.08)  (If indifferent)  0.49 (0.09)**  (If ambivalent) | 0.29 (0.12)*  (If aversive)  0.22 (0.07)*  (If indifferent)  -0.05 (0.15)  (If ambivalent) | 0.54 (0.03)**  (If high) | 0.43 (0.04)**  (If no conflict) | - |
|  | | | **Aus** | | | | | | | | |
| Trial if KIND Challenge | | | 0.00 (0.02) | 0.00 (0.11) | 0.06 (0.13) | 0.04 (0.07) | -0.18 (0.08)* | -0.02 (0.09) | 0.04 (0.04) | -0.05 (0.05) | 0.05 (0.04) |
| Age mean | | | 0.00 (0.03) | 0.04 (0.12) | 0.10 (0.19) | 0.12 (0.08) | 0.05 (0.09) | -0.01 (010) | 0.10 (0.05)* | -0.06 (0.06) | 0.08 (0.04)† |
| Gender (compared to female) | | |  |  |  |  |  |  |  |  |  |
|  | If male | | -0.01 (0.02) | 0.04 (0.11) | 0.02 (0.11) | 0.11 (0.07)† | 0.08 (0.08) | -0.03 (0.10) | -0.06 (0.04) | 0.09 (0.05)† | 0.02 (0.04) |
|  | If other | | 0.03 (0.03) | 0.02 (0.00)** | 0.52 (2.31) | -0.00 (0.07) | 0.06 (0.05) | -0.28 (2.42) | 0.02 (0.03) | 0.01 (0.05) | -0.25 (0.05)** |
| Length of time in Nextdoor (compared to <6 months) | | |  |  |  |  |  |  |  |  |  |
|  | If 1 year | | -0.02 (0.02) | 0.24 (0.12)* | 0.19 (0.32) | 0.03 (0.07) | -0.02 (0.09) | -0.08 (0.12) | 0.05 (0.04) | -0.01 (0.05) | 0.00 (0.04) |
|  | If over year | | -0.04 (0.03) | 0.04 (0.11) | 0.07 (0.15) | 0.03 (0.07) | 0.06 (0.09) | 0.01 (0.10) | 0.04 (0.04) | 0.10 (0.06)† | 0.01 (0.04) |
| Baseline loneliness | | | -0.08 (0.04)† | -0.45 (0.18)* | -0.31 (0.52) | 0.07 (0.12) | -0.08 (0.14) | 0.02 (0.16) | -0.02 (0.07) | -0.13 (0.09) | 0.10 (0.06)† |
| Baseline social isolation | | | 0.09 (0.04)* | -0.12 (0.17) | -0.10 (0.23) | -0.17 (0.11) | -0.20 (0.13) | -0.29 (0.25) | 0.01 (0.07) | -0.07 (0.08) | 0.26 (0.06)** |
| Baseline depression | | | -0.05 (0.03) | -0.23 (0.15) | -0.20 (0.35) | 0.14 (0.10) | 0.23 (0.11) | 0.07 (0.14) | 0.02 (0.06) | -0.08 (0.06) | -0.02 (0.06) |
| Baseline social anxiety | | | 0.03 (0.03) | 0.24 (0.14)† | 0.23 (0.39) | -0.12 (0.09) | -0.1 (0.10) | -0.14 (0.15) | -0.02 (0.05) | -0.02 (0.06) | 0.04 (0.05) |
| Baseline Neighborhood social cohesion and trust | | | 0.67 (0.03)** | 0.32 (0.13)* | 0.44 (0.70) | -0.08 (0.09) | -0.40 (0.11) | -0.28 (0.24) | 0.13 (0.05)* | 0.14 (0.06)* | 0.24 (0.05)** |
| Baseline number of Neighborhood contacts (if 6+) | | | 0.04 (0.03) | 0.01 (0.12) | -0.09 (0.18) | 0.15 (0.15) | -0.31 (0.19) | -0.53 (0.45) | 0.10 (0.05)* | -0.11 (0.06)† | 0.42 (0.04)** |
| COVID-19 related perceived restrictions | | | -0.03 (0.02) | 0.03 (0.10) | 0.06 (0.14) | -0.05 (0.06) | 0.07 (0.08) | 0.07 (0.10) | -0.01 (0.04) | 0.07 (0.05) | 0.01 (0.04) |
| Respective baseline outcome score | | | - | 0.47 (0.16)* (If stable)  0.87 (0.14)**  (If improving) | 0.69 (1.09)  (If stable)  0.58 (0.93)  If improving) | 0.68 (0.09)**  (If aversive)  -0.02 (0.10)  (If indifferent)  0.72 (0.08)**  (Ifambivalent) | 0.69 (0.07)**  (If aversive)  0.26 (0.07)**  (If indifferent)  0.39 (0.09)**  (If ambivalent) | 0.59 (0.43)  (If aversive)  0.44 (0.33)  (If indifferent)  0.22 (0.20)  (If ambivalent) | 0.51 (0.03)**  (If high) | 0.41 (0.04)**  (If no conflict) | - |
|  | | | **UK** | | | | | | | | |
| Trial if KIND Challenge | | | 0.03 (0.02) | 0.15 (0.09)† | 0.03 (0.09) | -0.06 (0.08) | -0.01 (0.08) | 0.08 (0.012) | 0.02 (0.04) | 0.06 (0.05) | -0.00 (0.04) |
| Age mean | | | -0.00 (0.03) | -0.13 (0.11 ) | -0.9 (0.10) | -0.01 (0.09) | 0.06 (0.10) | -0.16 (0.13) | 0.04 (0.05) | -0.01 (0.05) | 0.09 (0.04)* |
| Gender (compared to female) | | |  |  |  |  |  |  |  |  |  |
|  | If male | | 0.03 (0.02) | 0.10 (0.10) | 0.10 (0.10) | -0.09 (0.08) | -0.17 (0.09)† | -0.34 (0.19)† | -0.05 (0.04) | 0.02 (0.05) | 0.06 (0.05) |
|  | If other | | 0.02 (0.01)† | 0.08 (1.07) | 0.43 (0.73) | -0.04 (0.08) | -0.30 (0.59) | -0.33 (1.54) | -0.12 (0.05)* | -0.04 (0.07) | -0.02 (0.06) |
| Length of time in Nextdoor (compared to <6 months) | | |  |  |  |  |  |  |  |  |  |
|  | If 1 year | | 0.04 (0.03) | 0.05 (0.10) | 0.10 (0.10) | 0.09 (0.08) | -0.02 (0.09) | 0.12 (0.12) | 0.04 (0.05) | -0.08 (0.05)† | 0.03 (0.05) |
|  | If over year | | 0.02 (0.02) | -0.04 (0.10) | -0.03 (0.09) | -0.10 (0.09) | 0.00 (0.09) | 0.19 (0.15) | 0.01 (0.05) | -0.05 (0.05) | 0.05 (0.04) |
| Baseline loneliness | | | -0.03 (0.04) | -0.03 (0.16) | 0.10 (0.15) | 0.43 (0.13)** | 0.21 (0.14) | 0.16 (0.21) | 0.19 (0.08)* | -0.11 (0.08) | 0.05 (0.07) |
| Baseline social isolation | | | 0.12 (0.04)** | 0.23 (0.15) | 0.25 (0.17) | 0.33 (0.13)* | -0.14 (0.14) | -0.13 (0.21) | 0.22 (0.07)* | -0.06 (0.08) | 0.26 (0.07)** |
| Baseline depression | | | 0.01 (0.03) | -0.13 (0.13) | -0.17 (0.13) | -0.12 (0.12) | -0.05 (0.11) | 0.07 (0.16) | -0.01 (0.06) | -0.05 (0.06) | 0.06 (0.06) |
| Baseline social anxiety | | | -0.02 (.03) | -0.11 (0.012) | -0.06 (0.11) | -0.1 (0.11) | -0.00 (0.11) | -0.04 (0.14) | 0.03 (0.05) | 0.02 (0.06) | 0.01 (0.06) |
| Baseline Neighborhood social cohesion and trust | | | 0.69 (0.03)** | 0.37 (0.11)** | 0.31 (0.14)* | -0.35 (0.11)* | -0.52 (0.13)** | -0.39 (0.22)† | 0.07 (0.05) | 0.06 (0.06) | 0.15 (0.05)* |
| Baseline number of Neighborhood contacts (if 6+) | | | 0.08 (0.03)* | -0.26 (0.11)* | -0.25 (0.13)* | 0.20 (0.19) | 0.11 (0.19) | -0.58 (0.39) | 0.13 (0.05)* | 0.02 (0.05) | 0.55 (0.03)** |
| COVID-19 related perceived restrictions | | | 0.03 (0.02) | 0.01 (0.10) | -0.08 (0.09) | 0.01 (0.08) | 0.05 (0.08) | 0.01 (0.11) | 0.00 (0.05) | -0.05 (0.05) | 0.01 (0.04) |

†sign .10, *sign .05, **sign .001

Table S10. Compliance and acceptability ratings across countries

| **Compliance** | **Range** | **USA**  *M(SD)* | **AUS**  *M(SD)* | **UK**  *M(SD)* |
| --- | --- | --- | --- | --- |
| Number of KIND Challenge activities completed | >1 | 3.01 (1.38) | 2.58 (1.32) | 2.78 (1.40) |
| **Acceptability Rating** |  |  |  |  |
| Connected | 1 – 10 | 7.10(2.44) | 6.67 (2.47) | 6.56 (2.50) |
| Meaningful | 1 – 10 | 7.21 (2.41) | 6.29 (2.53) | 6.19 (2.74) |
| Safety | 1 – 10 | 8.90 (1.66) | 8.72 (1.81) | 8.69 (1.82) |
| Positive Feelings | 1 – 10 | 8.16 (1.93) | 7.51 (2.08) | 7.55 (2.15) |
| Comfortable | 1 – 10 | 7.52 (2.36) | 7.34 (2.42) | 7.10 (2.43) |

1. Dong N, Maynard R. PowerUp!: A tool for calculating minimum detectable effect sizes and minimum required sample sizes for experimental and quasi-experimental design studies. *J Res on Edu Eff*. 2013;6(1):24-67. [doi:10.1080/19345747.2012.673143](https://doi.org/10.1080/19345747.2012.673143) [↑](#footnote-ref-1)
